# Whole‐exome sequencing identified first homozygous frameshift variant in the *COLEC10* gene in an Iranian patient causing 3MC syndrome type 3

**DOI:** 10.1002/mgg3.1834

**Published:** 2021-10-12

**Authors:** Pouria Mohammadi, Elham Salehi Siavashani, Mohammad Farid Mohammadi, Afshin Bahramy, Navid Almadani, Masoud Garshasbi

**Affiliations:** ^1^ Department of Medical Genetics Faculty of Medical Sciences Tarbiat Modares University Tehran Iran; ^2^ PardisGene Co. Tehran Iran; ^3^ Department of Stem Cells and Developmental Biology Cell Science Research Center Royan Institute for Stem Cell Biology and Technology ACECR Tehran Iran; ^4^ Department of Cell and Molecular Sciences Faculty of Biological Sciences Kharazmi University Tehran Iran; ^5^ Department of Genetics Reproductive Biomedicine Research Center Royan Institute for Reproductive Biomedicine ACECR Tehran Iran

**Keywords:** 3MC syndrome type 3, c.128_129delCA, *COLEC10*, p.(Thr43AsnfsTer9)

## Abstract

**Background:**

3MC syndrome type 3 is an autosomal recessive disorder caused by mutations in the *COLEC10* gene besides other genes like *COLEC11* and *MASP1*. This disorder is characterized by facial dysmorphism, cleft lip and palate, postnatal growth deficiency, cognitive impairment, hearing loss, craniosynostosis, radioulnar synostosis, genital and vesicorenal anomalies, cardiac anomalies, caudal appendage, and umbilical hernia.

**Methods:**

In the present study, whole‐exome sequencing was performed in order to identify disease causing variant in an Iranian 7‐year‐old affected girl with craniosynostosis, dolichocephaly, blepharoptosis, clinodactyly of the 5th finger, high myopia, long face, micrognathia, patent ductus arteriosus, downslanted palpebral fissures, telecanthus, and epicanthus inversus. Identified variant confirmation in the patient and segregation analysis in her family were performed using Sanger sequencing method.

**Results:**

A novel homozygous frameshift deletion variant [NM_006438.5: c.128_129delCA; p.(Thr43AsnfsTer9)] was identified within the *COLEC10* gene. Up to now, only three 3MC syndrome patients with mutations in the *COLEC10* gene have been reported, and here, we report the fourth patient and the first homozygous frameshift variant.

**Conclusion:**

Other genes and factors responsible for 3MC syndrome occurrence are remained to be discovered. We believe further investigation of the genes in the lectin complement pathway is needed to be done for the identification of other causes of this disease.

## Graphical abstract

Up to now, only three 3MC syndrome patients with mutations in the *COLEC10* gene have been reported, and here, we report the fourth patient and the first homozygous frameshift mutation.

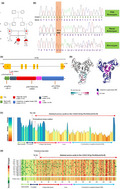

## 1 INTRODUCTION

3MC syndrome is a rare developmental disorder, which accounts for four separate autosomal recessive conditions with considerable overlapping clinical features, previously known as Mingarelli, Malpuech, Michels, and Carnevale syndromes (Gardner et al., 2017). 3MC syndrome is characterized by variable developmental anomalies including craniosynostosis, unilateral or bilateral cleft lip and/or palate, dysplastic ears, hypertelorism, blepharoptosis, downslanting palpebral fissures, postnatal growth retardation, hearing loss, skeletal anomalies, cognitive impairment, and other less common features, such as cardiac anomalies, hypospadias, and omphalocele (Urquhart et al., 2016). These phenotypes are probably due to defects in the induction, differentiation, and migration of cranial neural crest cells (CNCC) during embryonic development (Rooryck et al., 2011; Urquhart et al., 2016).

This syndrome can be caused by mutations in genes involved in lectin complement pathway comprised of *MASP1* (OMIM# 600521), *COLEC11* (OMIM# 612502), or *COLEC10* (OMIM# 607620) genes (Atik et al., 2015; Munye et al., 2017). Studies have shown that the lectin complement pathway components are integrated in the migration of CNCCs during embryonic development, in order to form several organs and tissues. Proper migration of CNCC lead to the morphogenesis of many facial and cranial tissues, hormone‐producing glands (endocrine glands), and parts of the nervous system. After birth, this pathway acts as a part of the innate immune system (Atik et al., 2015; Munye et al., 2017).

Located on chromosome 3q27‐28, the *MASP1* gene has three alternative splice products: MASP1, MASP‐3, and MAp44. The MASP‐1 and MASP‐3 have serine protease domain, while no serine protease domain is present in the MAp44 (Atik et al., 2015). Mutations in the *MASP1* gene, which encodes mannan‐binding lectin serine protease 1 (also known as mannose‐associated serine protease 1), are associated with 3MC syndrome type 1 (OMIM# 257920; Rooryck et al., 2011). The MASP1 protein, as a key component of the lectin complement pathway, recognizes and neutralizes pathogens (Rooryck et al., 2011).

Collectins, as other members of the innate immune system, have a carbohydrate recognition domain (CRD) and a collagen‐like region in their structures. The more well‐known parts of this group are collectin liver 1 (CL‐L1, also known as CL‐10), and collectin kidney 1 (CL‐K1, also known as CL‐11), which are encoded by *COLEC10* and *COLEC11* genes, respectively (Selman & Hansen, 2012). CL‐K1 protein is present in the serum, while CL‐L1 protein is restricted to the cytosol of hepatocyte cells. By binding to antigens such as bacteria, fungi, and viruses through CRDs and also interacting with MASP proteins, CL‐L1, and CL‐K1 are able to activate the lectin complement pathway (Munye et al., 2017). Mutations in the *COLEC11* gene, located on chromosome 2p25.3, reduce the normal serum level of CL‐K1 and possibly disrupt its interaction with MASP and CL‐L1. This defect results in 3MC syndrome type 2 (OMIM# 265050), which is clinically indistinguishable from 3MC syndrome type 1 (Selman & Hansen, 2012).

In 2017, Munye and colleagues, identified three compound heterozygous mutations [p.(Arg9Ter), p.(Gly77GlufsTer66), p.(Cys176Trp)] in *COLEC10*, as a novel gene in three Pakistani patients with 3MC syndrome (Munye et al., 2017). Through functional studies and gene expression evaluation in the mouse embryo, they demonstrated that mutations in the *COLEC10* gene and CL‐L1 deficiencies as a result are consistent with 3MC syndrome (Munye et al., 2017). Online Mendelian Inheritance in Men (OMIM) classifies this syndrome as 3MC syndrome type 3 (OMIM# 248340).

To the best of our knowledge, only three 3MC syndrome patients with mutations in the *COLEC10* gene have been reported, all of whom were compound heterozygous. In the current study, we described the fourth patient with 3MC syndrome type 3 and the first patient with homozygous frameshift variant in this gene.

## 2 MATERIALS AND METHODS

### 2.1 Ethical compliance

Written consent was obtained from parents as legal guardian of the proband. The study was performed in accordance with the ethical standards laid down in the 1964 Declaration of Helsinki and its later amendments (Shrestha & Dunn, 2019).

### 2.2 Whole‐exome sequencing and bioinformatics analysis

The patient's genomic DNA was extracted from her whole blood sample by applying salting‐out standard protocol. The Agilent SureSelect Human All Exon V7 Kit was used to enrich human whole exome. The generated library was sequenced on the NovaSeq 6000 platform with the mean coverage of 100X and eight Gb data were generated. An in‐house bioinformatics pipeline, Pardis Gene Exomine Technology, was used for bioinformatics analysis including the following steps:

1. Performing quality control (QC) of read length and depth based on GC content (about 50%), Phred value (Phred = 20) to find errors generated during the library preparation and sequencing. We performed this step utilizing the FastP tool which produces an HTML format QC report (Chen, Zhou, Chen & Gu, 2018).
2. Mapping the sequenced reads to the latest human reference genome (GRCh38/hg38) by Burrows–Wheeler aligner (BWA‐MEM). The Sequence Alignment/Map (SAM) was generated.
3. Converting SAM file to BAM file using Samtools (Li et al., 2009). BAM and BAM index file were used for the visualization of the read depth and variants localization by integrated genome viewer (IGV) software (Etherington, Ramirez‐Gonzalez & Maclean, 2015; Freese, Norris & Loraine, 2016).
4. Calling germline single nucleotide polymorphism (SNP) and insertion/deletion (Indel) variants was performed through genome analysis toolkit haplotypecaller (GATK HC). The output of this step was variant call format (VCF) file, which contained both SNP and Indel variants (Ren, Ahmed, Bertels & Al‐Ars, 2019).
5. Annotating VCF was done by Ensembl variant effect predictor (VEP; McLaren et al., 2016).
6. Filtering out was applied on the patient's variants. In this regard, common variants based on population databases including the genome aggregation database (gnomAD v3), the Exome aggregation consortium (ExAC v1) and Iranome (http://www.iranome.ir/), benign annotated ones, considering American college of medical genetics (ACMG) rules, and clinical databases such as the human gene mutation database (HGMD) professional, and ClinVar (https://www.ncbi.nlm.nih.gov/clinvar/) were excluded from the analysis. Then, based on a similar study (Mohammadi, Heidari, Ashrafi, Mahdieh & Garshasbi, 2021) by paying attention to the patient's clinical information provided to us, the human phenotype ontology (HPO) terms were extracted from the HPO as the most current reliable phenotype‐gene association database, in order to find related variants with the disorder. Moreover, OMIM as the most valid gene‐disease association database, was used to find the related disease with the altered gene in the patient (Mohammadi et al., 2020).

### 2.3 Family segregation study and protein analysis

At the first step, genomic DNA of the patient's both parents were extracted. The proband's mother was pregnant at the time of the analysis, so the fetus's DNA was extracted as well. The region including the detected variant in the *COLEC10* gene was PCR‐amplified considering the standard protocol. At the second step, primers using PrimerQuest tool and UCSC genome browser blat tools (Forward primer: TTCTGGGAGACCCTTTTCTG, Reverse primer: GGGAGGAAACTAGGGAGGAG), were designed and their quality and features were examined by OligoAnalyzer Tool (Rosenbloom et al., 2015). Third, confirmation of the identified variant in the proband and segregation study in her family by Sanger sequencing was done, using Applied Biosystems 3130 Genetic Analyzer, and Codoncode aligner software for the analysis of Sanger sequencing results. Functional domains, and regions of the COLEC10 protein were identified by UniProt (https://www.uniprot.org/). ConSurf server (https://consurf.tau.ac.il/), and PYMOL software were used in order to design 3D structural of the protein (Ashkenazy et al., 2016; Research & U. C., 2019). MetaDome was used to identify the intolerant regions in the COLEC10 protein (https://stuart.radboudumc.nl/metadome). Also, analysis of possible variants in the COLEC10 protein which is provided by PMut server (http://mmb.irbbarcelona.org/PMut/).

## 3 RESULTS

### 3.1 Clinical findings

In this study, we described an Iranian 7‐year‐old affected girl from consanguineous family, who was born with an uneventful pregnancy and delivery at 39th weeks of gestation. Her birth weight and height were normal, and she was admitted to the neonatal intensive care unit (NICU) on day 12 afterbirth due to peripheral cyanosis and respiratory insufficiency. Clinical examination, presented craniosynostosis at birth, therefore, first cranioplasty was performed at 5 months old and second one at 18 months old. At 1 year old, angiography was performed because of congenital heart defect (CHD). At 5 years old, additional clinical manifestations were dolichocephaly, blepharoptosis, blepharophimosis, telecanthus, epicanthus inversus, hypertelorism, downslanting palpebral fissure clinodactyly of the 5th finger, joint laxity, pes planus, loose skin, high myopia, long face, micrognathia, and patent ductus arteriosus (PDA). Height, intelligence, and metabolic tests (including complete blood count (CBC), fasting blood sugar (FBS), liver enzymes activity, low‐density lipoprotein (LDL), high‐density lipoprotein (HDL) were all in the normal range. The mother had a history of spontaneous abortion, also she had a therapeutic abortion at 17 weeks of pregnancy. The fetus was female and homozygous mutant for the c.128_129delCA variant in the *COLEC10* gene, which revealed by prenatal diagnosis (PND), using chorionic villus sampling (CVS).

### 3.2 Exome results

Prioritization and filtering of the most relevant variants, were manually assessed in order to detect those had the matched clinical outcomes and observed in the family by the following steps:

- Variants with minor allele frequency (MAF) >0.01 were filtered based on population databases including gnomAD v3, ExAC v1, and Iranome. Purpose: achieving rare variants in the patient. Remaining variants: 9,311 (Fattahi et al., 2019; Koch, 2020).
- Benign annotated variants by ACMG recommendations, and clinical databases consisting HGMD Professional and ClinVar, were excluded. Purpose: finding pathogenic variants in the patient. Remaining variants: 7,412 (Richards et al., 2008).
- By considering the patient's provided clinical and para‐clinical findings, HPO terms were extracted from the HPO database, as the most valid phenotype‐gene association database. Genes related to the following HPO terms were included in the analysis: craniosynostosis (HP:0001363), dolichocephaly (HP:0000268), ptosis (HP:0000508), clinodactyly of the 5th finger (HP:0004209), high myopia (HP:0011003), long face (HP:0000276), micrognathia (HP:0000347), patent ductus arteriosus (HP:0001643), downslanted palpebral (HP:0000494), telecanthus (HP:0000506), epicanthus inversus (HP:0000537). Purpose: finding variants related to the patient's phenotypes. Remaining variants: 39 (Köhler et al., 2019).
- The 39 remaining variants were analyzed based on ACMG recommendations. Disease associated with each gene bearing pathogenic alteration was studied in the OMIM database. Purpose: investigating the disease associated with the patient's phenotypes.
- Finally, the read length and the blat score of the final variant was checked using IGV software. Purpose: finding the mean coverage of the detected pathogenic variant.

**FIGURE 1:**
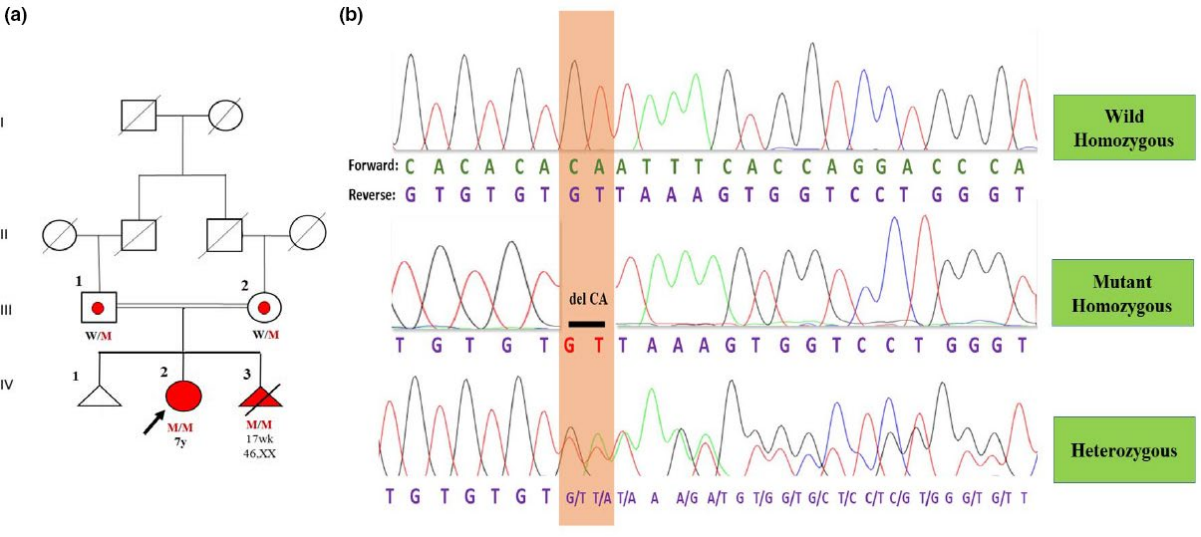
(a) Family pedigree of a 7‐year‐old affected girl offspring of consanguineous parents. As shown in the pedigree parents are first cousin. The affected proband is shown by arrow. The genotype of the parents and their affected girl for the c.128_129delCA variant is depicted on the pedigree. (b) Sanger sequencing chromatographs which shows homozygous frameshift variant in the patient, and affected fetus and heterozygous frameshift variant in the parents

In consequence, a novel homozygous frameshift deletion variant in the *COLEC10* gene [NM_006438.5: c.128_129delCA; p.(Thr43AsnfsTer9)] was identified, which classified as pathogenic (class 1) based on the ACMG classification. Pathogenic variants in the *COLEC10* gene are associated with autosomal recessive 3MC syndrome type 3 (Table 1; Munye et al., 2017). We confirmed the homozygous state for this variant in the proband and aborted fetus, as well as the heterozygote state in the parents by Sanger sequencing. Thereby, the c.128_129delCA variant in the *COLEC10* gene segregated with the 3MC syndrome type 3 in this family, and can be identified as cause of the disease in the proband (Figure 1).

**TABLE 1:** Detailed features of the identified variant based on online databases

Gene	Variant coordinates	Zygosity	Allele Frequencies^a^	Type and classification^b^	ACMG rules	Average coverage (X)	% Target bp covered^c^
*COLEC10*	Chr8 (hg38): g119067400 rs749987061 NM_006438.5: c.128_129delCA (p. Thr43AsnfsTer9) Exon1/6	Homozygous	gnomAD: 0.00001396 ExAC: 0.00002480 Iranome: NA	Frameshift Pathogenic (Class 1)	PVS1 PM2 PP4	87.17	0X: 1.73 1X: 98.27 2X: 98.12 10X: 97.76 20X: 97.46 50X: 89.45

^a^ Genome Aggregation Database (gnomAD) Genome version:3.0, Exome Aggregation Consortium (ExAC) version:1.0, and Iranome.

^b^ Variant classification is based on ACMG recommendations: Class 1: Pathogenic, Class 2: Likely pathogenic, Class 3: Variant of uncertain significance (VUS), Class 4: Likely benign, Class 5: Benign.

^c^ % Target bp Covered: the percentage of the covered target sequences based on the Agilent SureSelect Human All Exon V7 Kit. 0X: the percentage of the nucleotides with 0 coverage, 1X: the percentage of the nucleotides with 1 coverage and more, 2X: the percentage of the nucleotides with 2 coverage and more, 10X: the percentage of the nucleotides with 10 coverage and more, 20X: the percentage of the nucleotides with 20 coverage and more, 50X: the percentage of the nucleotides with 50 coverage and more.

## 4 DISCUSSION

For the first time, Munye et al. (2017), revealed that compound heterozygous pathogenic variants in the *COLE10* gene, cause autosomal recessive 3MC syndrome type 3. In a study (Munye et al., 2017), they evaluated 43 families of Asia, Middle Eastern and Europe origin, with 3MC syndrome type 3, using WES and Sanger sequencing methods. Mutation in *COLEC11* and *MASP1* genes was found in 30 families, including 32 patients. Also in two Pakistani families with three patients, WES identified three mutations with compound heterozygous manner in the *COLEC10*, as a novel gene, including a nonsense and a frameshift variant in an affected sibling [NM_006438.5: c.25C>T; p.(Arg9Ter) and NM_006438.5: c.226delA; p.(Gly77GlufsTer66)], and a nonsense and a missense variant in an affected girl [NM_006438.5: c.25C>T; p.(Arg9Ter) and NM_006438.5: c.528C>G;p.(Cys176Trp)]. The p.(Arg9Ter) and p.(Gly77GlufsTer66) are loss of function variants, which lead to null allele, p.(Cys176Trp) affects the disulfide bond between Cys176 and Cys270, which probably disrupts normal folding and secretion of the protein. Validation of the identified variants and segregation analysis in the Pakistani families was confirmed by Sanger sequencing. In addition, they observed expression of *COLEC10* in the cytosol and Golgi apparatus of murine chondrocyte cell line. Also, *COLEC10* expression in the epithelium and mesenchyme of the palate shelf and jaw in embryos was detected by expression analysis during murine craniofacial development. Interestingly, they did not find any mutation in the remained 28 families, which means pathogenic genomic alterations in the *MASP1*, *COLEC11*, and *COLEC10* genes are responsible for the minority of affected individuals with 3MC syndrome phenotypes, and the other genes and factors responsible for this disease occurrence are remained to be discovered (Munye et al., 2017).

Here, we described a seven‐year‐old girl from an Iranian consanguine family with craniosynostosis, dolichocephaly, blepharoptosis, blepharophimosis, telecanthus, epicanthus inversus, clinodactyly of the 5th finger, high myopia, long face, micrognathia, patent ductus arteriosus (PDA), and downslanting palpebral fissure. The clinical symptoms in our case seem to be more severe than Pakistani patients reported by Munye and colleagues (Munye et al., 2017). Craniosynostosis, dolichocephaly, high myopia, long Face, micrognathia, PDA, and downslanting palpebral fissure are not reported in any of them (Table 2).

**TABLE 2:** Comparison of clinical features and identified variants in the *COLEC10* gene for all 3MC three syndrome described patients

	Our case	Patient 1	Patient 2	Patient 3
Sex/age (year)	Female	Female	Male	Female
Origin	Iran	Pakistan	Pakistan	Pakistan
Mutated gene	*COLEC10*	*COLEC10*	*COLEC10*	*COLEC10*
cDNA change	c.128_129delCA	c.25C>T; c.226delA	c.25C>T; c.226delA	c.25C>T; c.528C>G
Amino acid change	p.(Thr43AsnfsTer9)	p.(Arg9Ter); p.(Gly77GlufsTer66)	p.(Arg9Ter); p.(Gly77GlufsTer66)	p.(Arg9Ter); p.(Cys176Trp)
Zygosity	Hom	Compound Het	Compound Het	Compound Het
Craniosynostosis	Y	N	N	N
Dolichocephaly	Y	N	N	N
Short stature	N	Y	Y	N
Belpharoptosis	Y	Y	Y	Y
High myopia	Y	N	N	N
Downslanting palpebral fissure	Y	N	N	N
Epicanthus inversus	Y	Y	Y	Y
Dysplastic ears and ears pit	N	N	N	Y
Micrognathia	Y	N	N	N
Long face	Y	N	N	N
Cleft lip and palate (unilateral)	N	N	Y	N
Cleft lip and palate (bilateral)	N	N	N	Y
Developmental delay	N	N	N	N
Polydactyly	N	N	Y	N
Clinodactyly	Y	N	N	Y
Patent Ductus Arteriosus	Y	N	N	N

Abbreviations: Het, heterozygous; Hom, homozygous; N, feature is not present; patient 1, patient 2, and patient 3 are all reported in one study (PMID: 28301481);Y, feature is present.

In this regard, a novel homozygous frameshift deletion variant, [NM_006438.5: c.128_129delCA; p.(Thr43AsnfsTer9)] was identified within the *COLEC10* gene, which creates a shift in the reading frame starting at codon 43. The new reading frame ends in a stop codon 9 positions downstream. This is the first time that a homozygous mutation has been reported in this gene.

**FIGURE 2:**
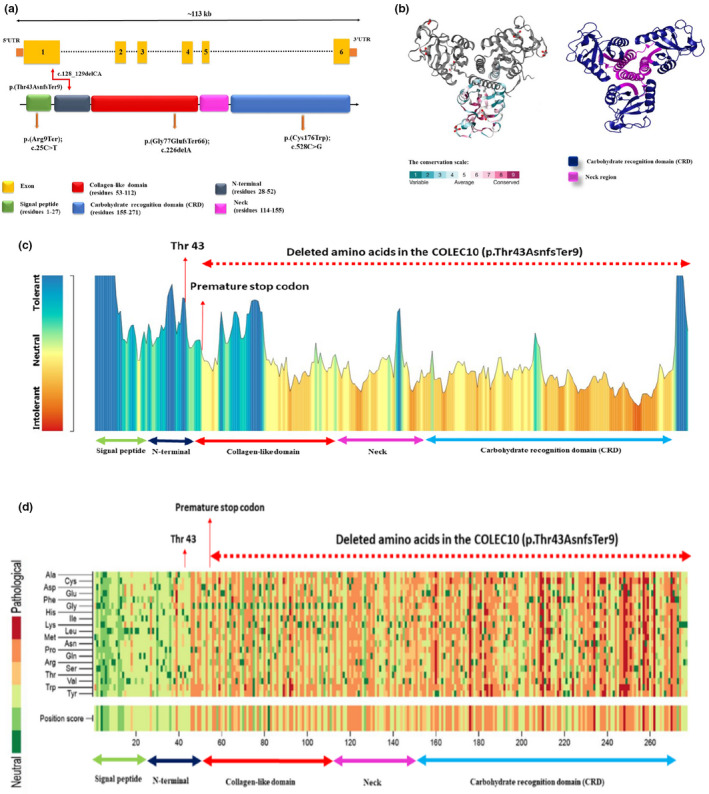
(a) The *COLEC10* gene contains six exons and spans ~113 kb. Graphical view of the COLEC10 protein (UniProtKB ‐ Q9Y6Z7) updated from UniProt (https://www.uniprot.org/uniprot/Q9Y6Z7). The canonical isoform length of the COLEC10 protein (identifier: Q9Y6Z7‐1) is 277 amino acids and contains two domains including collagen‐like domain (residue 52–112), and carbohydrate recognition domain (155–271). The p.(Thr43AsnfsTer9) variant is in the residue 43 of signal peptide region. (b) Three‐dimensional structure of the *COLEC10* protein provided by the ConSurf server and PyMOL software. (c) MetaDome (https://stuart.radboudumc.nl/metadome) was used to identify the intolerant regions in the COLEC10 protein. (d) Analysis of possible variants in the COLEC10 protein which is provided by PMut server (http://mmb.irbbarcelona.org/PMut/)

The *COLEC10* gene (OMIM# 607620), which spans 3133 bp, has six exons and only one protein coding transcript (ENST00000332843.3). Highly expressed in liver, moderately in lung, and weakly in artery coronary and adrenal gland, the Collectin‐10 protein has one isoform with 277 amino acids (Uniprot ID: Q9Y6Z7‐1), which contains a signal peptide (residue 1–27), a collagen‐like domain (residue: 53–112), a C‐type lectin domain (residue: 155–271), two disulfide bond (between Cys176‐Cys270 and Cys248‐Cys262), and a glycosylation site (Asn258; Figure 2; Selman & Hansen, 2012).

The *COLEC10* and *COLEC11* genes encode CL‐L1 and CL‐K1 proteins, respectively. The polypeptide chains (one of CL‐L1 and two of CL‐K1) join to form heterocomplexes called CL‐LK, which binds to *MASP 1*/*3* and *MASP 2* genes in the lectin complement pathway (Henriksen et al., 2013). In this way, CL‐LK complex by its chemotactic properties participates in the neural crest cells (NCCs) migration in the early stages of embryogenesis. The proper migration of NCCs is crucial for the formation of bone, cartilage, ganglia, and muscles in the head (Kitazawa et al., 2015; Minoux & Rijli, 2010). Mutations in this complex and specifically the frameshift deletion variant in the *COLEC10* gene in the present case, which cause premature stop codon and harbor the gene for nonsense‐mediated decay, resulting in not secreting and also not generating the protein product (Rooryck et al., 2011). In this regard, NCCs adhesion and migration are affected and there is an increase in NCCs dispersion. As a result, the affected individuals with 3MC syndrome show craniofacial conditions (Jani et al., 2016). In this study, we could find a frameshift deletion variant in the *COLEC10* gene in the patient showing 3MC syndrome phenotypes. We believe further investigation of the genes in the lectin complement pathway is needed to be done for the identification of other causes of this disease.

## CONFLICT OF INTEREST

The authors have declared no conflict of interest.

## AUTHOR CONTRIBUTIONS

MG conceived and designed the experiments. PM, ESS, NA, AB, and MFM conducted the experiments. MG, PM, ESS, and AB analyzed and interpreted the data. MG and PM contributed reagents/ materials/analysis tools. PM, ESS, MFM, and AB wrote the paper. All authors reviewed the manuscript.

## ETHICAL APPROVAL

The research protocol was approved by the ethics committee of Tarbiat Modares University, Tehran, Iran. The written informed consent was received from each guardian and they also provided a signed written consent to publish all personal and medical details included in this study.

## Data Availability

Human variant and pertinent phenotypes have been reported to ClinVar (Submission ID: SUB8558786, Accession ID: SCV001443819). https://submit.ncbi.nlm.nih.gov/subs/clinvar_wizard/SUB8558786/assertion_criteria.
